# Factors Associated with Exclusive Breastfeeding in Timor-Leste: Findings from Demographic and Health Survey 2009–2010

**DOI:** 10.3390/nu6041691

**Published:** 2014-04-22

**Authors:** Vishnu Khanal, Jonia Lourenca Nunes Brites da Cruz, Rajendra Karkee, Andy H. Lee

**Affiliations:** 1 Sanjeevani College of Medical Sciences, Butwal, Rupandehi, Nepal; E-Mail: khanal.vishnu@gmail.com; 2 National Hospital Guido Valadares, Ministry of Health, Dili, Timor Leste; E-Mail: joniadacruz@yahoo.com; 3 School of Public Health and Community Medicine, BP Koirala Institute of Health Sciences, Dharan, Nepal; E-Mail: rkarkee@yahoo.com; 4 School of Public Health, Curtin University, Perth, WA 6153, Australia

**Keywords:** breastfeeding, demographic and health survey, exclusive breastfeeding, Timor-Leste

## Abstract

Exclusive breastfeeding is known to have nutritional and health benefits. This study investigated factors associated with exclusive breastfeeding among infants aged five months or less in Timor-Leste. The latest data from the national Demographic and Health Survey 2009–2010 were analyzed by binary logistic regression. Of the 975 infants included in the study, overall 49% (95% confidence interval 45.4% to 52.7%) were exclusively breastfed. The exclusive breastfeeding prevalence declined with increasing infant age, from 68.0% at less than one month to 24.9% at five months. Increasing infant age, mothers with a paid occupation, who perceived their newborn as non-average size, and residence in the capital city Dili, were associated with a lower likelihood of exclusive breastfeeding. On the other hand, women who could decide health-related matters tended to breastfeed exclusively, which was not the case for others whose decisions were made by someone else. The results suggested the need of breastfeeding promotion programs to improve the exclusive breastfeeding rate. Antenatal counseling, peer support network, and home visits by health workers could be feasible options to promote exclusive breastfeeding given that the majority of births occur at home.

## 1. Introduction

Exclusive breastfeeding means only breastmilk is allowed with the exception of medicine, vitamin syrup and oral rehydration solution [1,2]. It is known that exclusive breastfeeding for six months can protect infants from short term illnesses such as gastroenteritis, respiratory infection and under nutrition; and in the long term, against chronic diseases such as type 2 diabetes, hypertension and obesity [3,4]. The economic benefits include cost savings from avoiding illness, workdays lost and the purchase of infant formula [5]. Moreover, it has been projected that 11.6% of child deaths in 2011 could be attributable to sub-optimal breastfeeding [6]. A variety of factors have been reported to affect the practice of exclusive breastfeeding, including maternal characteristics (education, occupation, health condition, age), infant characteristics (sex, birth order, illness), and cultural practices (initiation of breastfeeding, time of introduction of complementary feeds) [7,8]. The effects of these factors vary according to cultural context and related socioeconomic conditions.

Timor-Leste is one of the youngest countries in Asia which gained independence since 2002 [9]. The country went through a long armed conflict during 1990s, leading to destruction of most of the infrastructure [9], leaving thousands of its citizens being displaced from East to West Timor [10]. The majority of health workforce returned to Indonesia after independence. Only a small number of health professionals remained to re-establish the health system with assistance from the United Nations, expatriate workers, and other international support [10]. The population of Timor-Leste is estimated to be 1.07 million in 2010 with a growth rate of 2.4% [11]. The country has a high infant mortality (45 per 1000 live births) and under five mortality rate (64 per 1000 live births). The proportion of under five children suffering from under-nutrition is still high: stunting (53%), wasting (17%) and under-weight (52%) [11]. Exclusive breastfeeding has been found positively associated with infant stature [12] and protective against overweight and obesity in childhood [13].

Currently, information on infant nutrition in Timor-Leste is lacking. A previous survey conducted in 2003 [14] reported that breastfeeding was almost universal (97.6%), but a much lower exclusive breastfeeding rate of 30.7% for infants less than 6 months. Some health infrastructures have been restored after independence in 2002. For instance, 5 hospitals, 69 health centers and 85 health posts have been established between 2002 and 2005. A national referral hospital and community health centers were also functioning by this time with the support from the government and international community. The per capita expenditure on health was US $45 in 2006 (7.5% of GDP), higher than many other Asian countries [9]. As part of the global measure Demographic and Health Surveys (DHS) project, an updated Timor-Leste DHS was conducted during 2009–2010 [11]. The aim of the present study was to investigate factors associated with exclusive breastfeeding among infants aged five months or less based on the updated information from the national DHS, findings from which will enable policy makers and public health researchers to develop interventions to improve exclusive breastfeeding in the country.

## 2. Methods

### 2.1. Survey Design

DHS are conducted every five years in more than 50 countries using a validated questionnaire. The Timor-Leste DHS 2009–2010, conducted in two stages, was the second survey after the initial 2003 DHS. During the first stage, 455 enumeration areas were selected based on probability proportionate to size: 116 urban and 339 rural areas. More rural samples were included because the majority of the Timor-Leste population lives in rural areas. At the second stage, 27 households were selected randomly from each enumeration area following a systematic sampling procedure.

### 2.2. Participants

The final survey included 11,463 households, comprising 9806 children under five years of age. The present study focused on the subgroup of 975 infants (1) with a singleton birth; (2) who were aged less than six months; (3) alive and living with the respondent; and (4) who were the youngest child in the family; in order to avoid the selection of children from the same household and parents. The DHS was approved by the ethics committee of Macro International Inc. and the Ministry of Health of Timor-Leste. The data were de-identified and made available for public use [15].

### 2.3. Exclusive Breastfeeding

The operational definition of exclusive breastfeeding, as defined by the World Health Organization (WHO) infant feeding guidelines [2], was adopted: “infants 0–5 months of age who are fed exclusively with breastmilk”. Apart from breastmilk or wet nurse’s milk, no other fluid was allowed, with the exception of oral rehydration solution, drops or syrups (vitamins, minerals and medicine). The binary status of exclusive breastfeeding (coded as ‘1’ for yes and ‘0’ for no) was determined for each of the 975 selected infants. Previous published studies have used such definition to report a period prevalence of exclusive breastfeeding based on 24-h recall [16,17], but it should not be treated as the rate of exclusive breastfeeding for six months.

### 2.4. Independent Variables

Selection and categorization of independent variables in this study were based on literature review [14,18]. Maternal age was recoded into three groups: 15–19, 20–34 and 35–49 years. Frequency of antennal care (ANC) visit was categorized as: 0, 1–3 and ≥4. Mother’s perceived size of newborn was coded as: small, average and large. Religion was originally recorded as: Roman Catholic, Muslim, Protestant, Hindu and others. Because the vast majority of population follows Roman Catholic, the other religions were grouped together. Maternal occupation was re-categorized as: no paid work (housewives and household work), agriculture (self-employed or employee), professional, clerical, sales and services, and manual work (skilled or unskilled). Education level was classified as: no education, primary, secondary and higher. Decision making on health had three categories: respondent alone, respondent with others (e.g., husband), others only (e.g., husband or someone else) [19]. Place of delivery was regrouped as either health facility (national hospital, referral hospitals, community health centers, health post, SisCa post, private sectors, Marrie stops) or home (home of respondent or others). Birth order referred to: first time birth, second or third, and fourth or above. Residential location was defined as either rural or urban.

### 2.5. Statistical Analysis

Timor-Leste is divided into 13 administrative districts, which has been incorporated into the analysis to examine geographical differences in the 24-h period prevalence of exclusive breastfeeding among infants aged <6 months. Further age-wise disaggregated proportions of exclusive breastfeeding were reported at age <1 month, 1, 2, 3, 4, and 5 months. Factors associated with exclusive breastfeeding were screened by Chi-square tests and then assessed by backward stepwise logistic regression [20], taking into account the apparent collinearity between independent variables. Complex sample analysis was performed to estimate proportions, odds ratios and their 95% confidence intervals (CI) [21].

## 3. Results

### 3.1. Exclusive Breastfeeding

As shown in Table 1, of the 975 infants aged ≤5 months, overall 49% (95% CI 45.4% to 52.7%) were exclusively breastfed in the 24 h preceding the 2009–2010 survey. The exclusive breastfeeding prevalence appeared to decline with increasing infant age, from 68.0% at less than one month to 24.9% at five months. In particular, a sharp decrease was observed between the 4th and 5th month postpartum for the respondents.

**Table 1:** Prevalence of exclusive breastfeeding by infant age, Timor-Leste, 2009–2010 (*n* = 975).

Infant Age (months)	*n*	Prevalence (95% Confidence Interval)
<1	80	68.0 (55.4, 78.5)
1	177	67.6 (59.2, 75.0)
2	151	56.5 (46.5, 66.1)
3	183	48.5 (40.5, 56.6)
4	209	41.8 (34.3, 49.7)
5	175	24.9 (19.5, 31.2)
≤5	975	49.0 (45.4, 52.7)

### 3.2. Sample Characteristics

The distribution of maternal age (years) was: 15–19 (6.8%), 20–34 (67.5%), 35–49 (25.7%). The majority (78.6%) of participants resided in rural areas. About one-third (34.6%) of mothers and just over a quarter (28.5%) of fathers received no education. The majority of women had no paid employment (73.2%) and followed Roman Catholic as their religion (98.2%). More of them came from poorer (45.4%) than middle class (39.6%) and rich households (15%). Most respondents did not read newspaper (70.3%), listen to the radio (59.9%), or watch television (68.6%) at all.

Slightly more than half (52.7%) of mothers had high parity (≥4), with only 17.4% being first time motherhood. Only 52% of the women had paid four or more ANC visits. There were more male (52.9%) then female (47.1%) infants. The majority of infants were born at home (74.2%) by vaginal delivery (98.3%). Surprisingly, only about a quarter (26.6%) of women could make decision by themselves with regard to health-related matters. Although 56% of mothers perceived their newborn as average size, 18.5% believed they were small size. Most mothers (88.8%) initiated breastfeeding within one hour of delivery. Only a small proportion (2.9%) of mothers continued to smoke at the time of the survey.

A number of socio-demographic and health-related variables appeared to be associated with the prevalence of exclusive breastfeeding according to Chi-square tests. These included residential location (*P* = 0.014), ecological region (*P* < 0.001), maternal occupation (*P* < 0.001), wealth status (*P* = 0.003), frequency of listening to the radio (*P* = 0.007), frequency of watching television (*P* = 0.004), and decision making on health (*P* = 0.026).

### 3.3. Factors Affecting Exclusive Breastfeeding

Stepwise logistic regression analysis further confirmed that infant age, ecological region, maternal occupation, perceived size of newborn, and decision making on health were significantly associated with exclusive breastfeeding; results of which are shown in Table 2. In particular, increase in infant age could reduce the likelihood of exclusive breastfeeding, consistent with the previous observation on the decline in exclusive breastfeeding prevalence by advancing infant age in Table 1. When compared to the capital city Dili, mothers from other regions were more likely to exclusively breastfeed their infants. On the other hand, mothers who maintained employment seemed less likely to continue exclusive breastfeeding than their non-working counterparts. Those mothers who perceived their newborn as either large or small size were also less likely to exclusively breastfeed. Finally, mothers who could decide health-related matters by themselves tended to exclusively breastfeed, which was not the case for others whose decisions were made by someone else.

## 4. Discussion

This study found that half (49.0%, 95% CI 45.4% to 52.7%) of the infants aged five months or below were exclusively breastfed at the time of the 2009–2010 DHS, which appeared to increase substantially from the previously reported 24-h recall prevalence rate of 30.77% (95% CI 27.2% to 34.5%) in 2003 [14]. According to the report by UNICEF [22], the proportion of exclusively breastfed children of 0–5 months during the period 2000–2007 was 43% in East Asia and Pacific, 44% in South Asia, and 39% overall in developing countries. However, the differences in survey period between countries should be taken into account. The apparent increase in exclusive breastfeeding prevalence may be attributable to a number of changes in Timor-Leste since 2003. The country has become more stable after the conflict, with social and health services being restored [9,11]. While it is encouraging to note the improvement in exclusive breastfeeding practice, the rate is still much lower than the recommended 90% by the WHO [23].

**Table 2:** Factors associated with exclusive breastfeeding in Timor-Leste, 2009–2010 (*n* = 975).

Factor	*n* (%)	EBF (%)	Adjusted Odds Ratio (95% Confidence Interval) *	*P* *
**Infant age (months)**	Mean 2.81	SD 1.58	0.67 (0.60, 0.71)	<0.001
**Ecological region**				<0.001
Dili (Capital)	86 (6.3)	30 (33.2)	1.00	
Aileu	83 (8.5)	61 (73.6)	8.05 (3.43, 18.89)	
Ainaro	73 (7.5)	49 (67.2)	5.10 (2.49, 10.43)	
Baucau	65 (6.7)	36 (52.3)	2.83 (1.23, 6.51)	
Bobonaro	78 (8.0)	30 (39.4)	1.23 (0.62, 2.44)	
Cova Lima	61 (6.3)	21 (34.0)	1.06 (0.51, 2.21)	
Ermera	102 (10.5)	52 (50.4)	2.21 (1.10, 4.44)	
Liquica	84 (8.6)	53 (63.3)	3.75 (1.90, 7.38)	
Lautem	72 (7.4)	35 (46.5)	1.93 (1.03, 3.64)	
Manufahi	63 (6.5)	39 (61.6)	3.80 (1.75, 8.28)	
Manatuto	81 (8.1)	34 (41.5)	1.44 (0.76, 2.74)	
Oecussi	84 (8.6)	50(59.5)	3.98 (2.21, 7.15)	
Viqueque	43 (4.4)	24 (55.3)	2.26 (2.21, 7.15)	
**Maternal occupation**				0.003
No paid work	712 (73.2)	394 (52.8)	1.00	
Agriculture	176 (18.1)	91 (46.1)	0.68 (0.46, 1.02)	
Professional, clerical, sales, services	67 (6.9)	23 (24.3)	0.33 (0.18, 0.62)	
Manual work	18 (1.8)	5 (34.4)	0.67 (0.19, 2.31)	
**Perceived size of newborn**				0.009
Average	536 (56.0)	301 (53.0)	1.00	
Small	177 (18.5)	90 (45.6)	0.61 (0.39, 0.93)	
Large	244 (25.5)	114 (43.2)	0.58 (0.38, 0.88)	
**Decision making on health**				0.023
Others only	106 (11.1)	43 (35.1)	1.00	
Respondent alone	254 (26.6)	143 (52.1)	2.02 (1.11, 3.67)	
Respondent with others	595 (62.3)	318 (50.6)	1.63 (0.89, 2.98)	

EBF: exclusive breastfeeding. * From backward stepwise logistic regression; variables excluded were: maternal age, residential location, maternal education, paternal education, religion, sex of infant, wealth status, frequency of reading newspaper/magazine, frequency of listening radio, frequency of watching television, birth order, frequency of antenatal care visit, maternal tobacco smoking, method of delivery, place of delivery.

The prevalence of exclusive breastfeeding declined with increasing infant age, from 68.0% at less than one month to 24.9% at five months. The inverse association between infant age and exclusive breastfeeding practice was also observed in other Asian countries such as Bangladesh, China and Nepal [18,24,25]. According to the local culture, it is common that Timorese infants are introduced complementary foods at about the 4th month. The decision is usually made by the senior women of the family such as the grandmother or grandmother-in-law.

Mothers residing in Dili were less likely to breastfeed exclusively when compared with mothers from other regions. Such regional differences have been reported by previous studies in Timor-Leste and other Asian countries [14,17]. Dili is the capital and economic center of the country, where infant formulas are readily accessible at supermarkets. Besides, the capital city citizens are more exposed to advertisement of infant formula, consequently leading to the early cessation of exclusive breastfeeding [26].

Moreover, women who maintained employment after giving birth were less likely to provide exclusive breastfeeding to their infants than their non-working counterparts. Similarly, Chinese mothers who had to return to their office job before six months were unlikely to breastfeed their infant exclusively [24]. Another qualitative study from Bangladesh reported that caretakers introduced formula, cow or buffalo milk when mothers attended work [27]. Working mothers in Timor-Leste are entitled to less than three months of maternity leave. This short duration makes it difficult to continue exclusive breastfeeding.

Newborns perceived to be non-average size by their mothers were less likely to be exclusively breastfed. Experience in other countries has similarly shown that preterm and low birth weight infants are breastfed for shorter duration [28]. Mothers may experience a number of barriers to breastfeed smaller infants, for instance, poor sucking, infants being kept separately for intensive care, illness, and lack of confidence [29], which may lead to the early introduction of complementary foods.

In this study, Timorese mothers who could decide health-related matters tended to continue exclusive breastfeeding, when compared with those that relied on the advice from someone else. This finding was consistent with the literature, which suggested that the ability of a woman to make decision on utilization of services can lead to better maternal and child health outcomes [19,30].

Several issues should be considered when interpreting the results. This study utilized the dataset from the latest national survey with a representative sample and a high response rate, while complex sample analysis was performed to account for the sampling strategy and sample weight [21]. Therefore, the findings are generalizable to the entire country. However, the 24-h recall would inevitably induce over-reporting of exclusive breastfeeding at six months [31] so that caution should be taken [2]. The DHS data nonetheless remain the only available information to estimate exclusive breastfeeding rate in many developing countries.

There is an immediate need of breastfeeding promotion programs in Timor-Leste. Given the high infant and child mortality in the country [11], improving the practice of exclusive breastfeeding will reduce such burden and partially overcome the problem of under-nutrition. Antenatal counseling on breastfeeding and peer support network are recommended [32]. Because the majority of births occur at home, home visits by health workers/volunteers would be an effective option to consider by healthcare planners to further promote exclusive breastfeeding and to increase its duration.

## 5. Conclusions

Slightly less than half the infants in Timor-Leste were exclusively breastfed within 24-h preceding the latest national survey. This represented a significant improvement in exclusive breastfeeding practice since 2003 when the country restored peace. Mothers should be provided with continuous support to sustain their initial high rate of exclusive breastfeeding for six months. It is desirable to target mothers who are working, who perceive their newborns as non-average size and those residing in the capital Dili for breastfeeding promotion programs. In addition, mothers must be involved in the decision making process so that they can sustain breastfeeding exclusively.
